# RETRACTION: Long Noncoding RNA UFC1 is Activated by E2F1 and Exerts Oncogenic Properties by Functioning as a CeRNA of FOXP3

**DOI:** 10.1002/cam4.70150

**Published:** 2024-10-01

## Abstract

**RETRACTION**: J. Xi, J. Feng, S. Zeng, and P. Huang, “Long Noncoding RNA UFC1 is Activated by E2F1 and Exerts Oncogenic Properties by Functioning as a CeRNA of FOXP3,” *Cancer Medicine* 7, no. 7 (2018): 3301–3310. https://doi.org/10.1002/cam4.1556.

The above article, published online on 23 May 2018 in Wiley Online Library (wileyonlinelibrary.com), has been retracted by agreement between the journal Editor‐in‐Chief, Stephen Tait; and John Wiley and Sons Ltd. The retraction has been agreed following an investigation into concerns raised by a third party, which revealed inappropriate duplications of image panels (Figure 3A–C, Figure 4C (HeLa–FOXP3 and GAPDH lanes) and D (FOXP3 lane)) between this and another article previously published by a different group of authors, in a different scientific context, elsewhere. Given the extent of the identified issues, the editors have lost confidence in the data presented and the article's conclusions can no longer be considered reliable. The authors have been informed of the allegations and the decision to retract but they remained unresponsive.

**RETRACTION**: J. Xi, J. Feng, S. Zeng, and P. Huang, “Long Noncoding RNA UFC1 is Activated by E2F1 and Exerts Oncogenic Properties by Functioning as a CeRNA of FOXP3,” *Cancer Medicine* 7, no. 7 (2018): 3301–3310. https://doi.org/10.1002/cam4.1556.

The above article, published online on 23 May 2018 in Wiley Online Library (wileyonlinelibrary.com), has been retracted by agreement between the journal Editor‐in‐Chief, Stephen Tait; and John Wiley and Sons Ltd. The retraction has been agreed following an investigation into concerns raised by a third party, which revealed inappropriate duplications of image panels (Figure 3A–C, Figure 4C (HeLa–FOXP3 and GAPDH lanes) and D (FOXP3 lane)) between this and another article previously published by a different group of authors, in a different scientific context, elsewhere. Given the extent of the identified issues, the editors have lost confidence in the data presented and the article's conclusions can no longer be considered reliable. The authors have been informed of the allegations and the decision to retract but they remained unresponsive.

